# Meat Quality and Fatty Acid Profile of Rams Fed Diets Enriched with Vegetable Oils of Varying Unsaturation Levels

**DOI:** 10.3390/foods14132179

**Published:** 2025-06-22

**Authors:** Evyla Layssa Gonçalves Andrade, Kevily Henrique de Oliveira Soares de Lucena, José Morais Pereira Filho, Marcia Makaline Rodrigues Pereira, Ronaldo Lopes Oliveira, Analívia Martins Barbosa, Elzania Sales Pereira, Claudio Vaz Di Mambro, Marcos Jacome de Araújo, Leilson Rocha Bezerra

**Affiliations:** 1 Graduate Program in Animal Science and Health Animal Science Department, Federal University of Campina Grande, Patos 58708-110, PB, Brazil; evylalayssa@hotmail.com (E.L.G.A.); hkevily@gmail.com (K.H.d.O.S.d.L.); jmorais@cstr.ufcg.edu.br (J.M.P.F.); marciamakaline@gmail.com (M.M.R.P.); 2 Animal Science Department, Federal University of Bahia, Salvador 40170-155, BA, Brazil; ronaldooliveira@ufba.edu (R.L.O.); analiviabarbosa@gmail.com (A.M.B.); claudioribeiro@ufba.br (C.V.D.M.); 3 Department of Animal Science, Federal University of Ceará, Mister Hull Avenue, Fortaleza 60356-000, CE, Brazil; elzania@hotmail.com; 4 Department of Animal Science, Federal University of Piaui, Bom Jesus 64900-000, PI, Brazil; jacome@ufpi.edu.br

**Keywords:** biohydrogenation, cashew, lipid, oil, oleic acid, ruminant

## Abstract

Antioxidant feed additives, such as cashew nutshell liquid (CNSL), rich in phenolic compounds, have gained attention for improving animal production and meat quality. The study evaluated the dietary effects of blending CNSL (0.5%) with different vegetable oils (1.5%) varying in unsaturated fatty acid (UFA) profiles on the meat quality and fatty acid composition of muttons. Forty Santa Inês × Dorper crossbred rams (24.44 ± 1.5 kg) were allocated to five treatments for 70 days: CNSL combined with canola (MUFA-rich) compared to corn, soybean, sunflower, or cottonseed oils (PUFA-rich). The CNSL + canola blend improved meat quality, showing higher water-holding capacity and tenderness and lower cooking loss than CNSL + PUFA-rich oils (*p* < 0.05). Meat yellowness (b*) increased with CNSL + soybean or cottonseed blend. Meat proximate composition was unaffected (*p* > 0.05). Minor variations in specific fatty acids were observed, including higher C14:0 and C16:1 in canola and cottonseed + CNSL blend groups and greater EPA (C20:5 n–3) with soybean oil + CNSL blend (*p* < 0.05). The ∑n–6:∑n–3 ratio was highest with cottonseed and lowest with soybean oil (*p* < 0.05). Overall, combining CNSL with MUFA-rich oils, particularly canola, compared to PUFA oils, enhances meat quality while supporting the sustainable use of CNSL in ram diets.

## 1. Introduction

Including vegetable oils in ruminant diets is an effective strategy to enhance the efficiency of metabolizable energy utilization for growth and finishing. This occurs by reducing ruminal fermentation and heat increment while modulating the fatty acid (FA) profile through ruminal biohydrogenation. Consequently, this alters the FA composition of animal-derived products such as meat [1,2] and milk [3], directly impacting their nutritional quality. This is particularly relevant considering the different effects that dietary fat, through its FA profile, can exert on human health [4,5].

Vegetable oils differ in their fatty acid composition, primarily due to genetic factors of the source plant, environmental conditions, and post-harvest processing [6]. Moreover, each plant species possesses a unique enzymatic profile that determines the proportion of synthesized fatty acids during oil formation in seeds, fruits, or pulp [7]. Commonly tested oils in ruminant diets include canola oil, which is rich in oleic acid (a monounsaturated fatty acid, MUFA); soybean and corn oils, which are high in linoleic acid (an omega-6 polyunsaturated fatty acid, PUFA); and flaxseed and sunflower oils, which are abundant in alpha-linolenic acid (an omega-3 PUFA) [8,9,10,11].

When ruminants are fed diets containing vegetable oils high in polyunsaturated fatty acids (PUFAs), such as soybean, corn, or sunflower oil, these unsaturated fatty acids (UFAs) enter the rumen, where microbial lipases promptly hydrolyze triglycerides into free fatty acids and glycerol [12]. The liberated glycerol is subsequently fermented by rumen microbes, producing volatile fatty acids (VFAs), primarily propionate, which serves as a key energy source for the animals [13]. Linoleic acid, one of the predominant PUFAs, undergoes isomerization at the cis-12 double bond, resulting in the formation of conjugated isomers—mainly rumenic acid (cis-9, trans-11 conjugated linoleic acid, CLA) [14,15]. Through a sequence of reductive reactions catalyzed by microbial enzymes, these conjugated intermediates are gradually hydrogenated, first yielding vaccenic acid (trans-11 C18:1) and finally stearic acid (C18:0), which represents the end product of the biohydrogenation pathway [16].

Stearic acid (C18:0) is the main end product of biohydrogenation and represents a saturated fatty acid that contributes substantially to the high SFA content found in ruminant meat [1,5,17]. However, when biohydrogenation is incomplete, certain intermediates, such as CLA and trans-11 vaccenic acid, can escape ruminal metabolism, be absorbed in the intestine, and influence the lipid composition of meat and milk, enhancing the content of functional compounds like CLA and omega-3 fatty acids [18,19].

In this context, the inclusion of compounds such as cashew nutshell liquid (CNSL) from Anacardium occidentale in ruminant diets may have beneficial effects on fat quality. CNSL has well-documented antioxidant and bactericidal properties, which can modulate ruminal fermentation patterns, reduce enteric methane emissions, improve animal performance, and enhance nutrient metabolism [20,21]. Previous studies have demonstrated that CNSL supplementation at levels of up to 0.5% of total dry matter improves the performance of sheep [5] and alters the fatty acid profile of meat [11,21]. CNSL is an agro-industrial by-product from cashew processing, classified as a phenolic lipid composed primarily of anacardic acid, cardanol, and cardol, long-chain unsaturated phenolic compounds with potential benefits for ruminant nutrition [22].

Given this context, we hypothesize that the inclusion of cashew nutshell liquid (CNSL) in diets containing polyunsaturated fatty acids (PUFAs), such as linoleic and alpha-linolenic acids, may offer partial protection of these fatty acids against ruminal biohydrogenation, thereby promoting greater passage to the intestine and enhancing their subsequent absorption. This could lead to increased health-promoting lipids in meat, including conjugated linoleic acid (CLA) and omega-3 fatty acids. Additionally, the combination of CNSL with monounsaturated fatty acids (MUFAs), such as oleic acid from canola oil, may help mitigate the inhibitory effects of unsaturated fats on rumen microbial populations, potentially improving nutrient digestion, animal performance, and intramuscular fat deposition. Therefore, this study aimed to determine the most effective combination of CNSL and unsaturated fatty acids from vegetable oils on meat quality parameters and the fatty acid profile of lamb meat.

## 2. Materials and Methods

### 2.1. Ethical Considerations, Diets, Animals, and Experimental Design

All animal handling and experimental procedures were conducted according to the ethical guidelines established by the Ethics Committee on Animal Use of the Federal University of Campina Grande (CEUA/UFCG-CSTR) under protocol approval number 55/2022.

The experimental diets were prepared as total mixed rations (TMRs) with a forage-to-concentrate ratio of 40:60, formulated to satisfy the nutritional demands for an expected average daily weight gain of 250 g/day, according to the National Research Council guidelines [23]. Sorghum silage served as the roughage component. The concentrate portion consisted of ground corn, soybean meal, a mineral mixture, and the experimental additives (Table 1), which included cashew nutshell liquid (CNSL) at 0.5% of the total dry matter (DM) in combination with vegetable oils at 1.5% DM.

The dietary lipid sources used in this study included canola oil, characterized by a high concentration of monounsaturated fatty acids (MUFAs), and corn, soybean, sunflower, and cottonseed oils, which are predominantly composed of polyunsaturated fatty acids (PUFAs). Animals were fed twice daily, at 08:00 and 15:00, and the amount of feed offered was adjusted to maintain approximately 10% refusals, ensuring ad libitum intake. Clean, fresh water was provided at all times. Daily dry matter (DM) intake, metabolizable energy, and individual fatty acids were estimated by calculating the difference between the nutrient content in the feed offered and the orts (refusals).

The cashew nutshell liquid (CNSL) used in this trial was obtained through thermal extraction. Initially, cashew shells were subjected to steam treatment at 80 °C, followed by mechanical pressing to extract the CNSL and generate a residual shell cake [24]. Subsequently, a solvent-free thermomechanical process was applied, and the previously extracted CNSL served as the heating medium. This method elevated the temperature of the nuts to approximately 190 °C, facilitating the rupture of the shell and the release of alkyl phenolic compounds from the mesocarp. This allowed for separating the inner shell and recovering the kernel [22]. After extraction, the CNSL was subjected to a decarboxylation step to eliminate moisture and carbon dioxide. This was achieved by heating the liquid to 140 °C under constant agitation [24]. During this process, anacardic acid is thermally converted into cardanol, resulting in “technical CNSL” [22], the form used in the present study. The final product was filtered using a pressurized filtration system and stored appropriately until its inclusion in the experimental diets.

Forty crossbred, intact male rams with an initial mean body weight of 24.44 ± 1.5 kg and an average age of 8 months were selected. Prior to the start of the trial, all animals were vaccinated against clostridial diseases and rabies and treated for both endo- and ectoparasites. The rams were individually housed in suspended pens equipped with individual feed bunks and water troughs. The study lasted for 70 days, comprising a 15-day adaptation period to the housing conditions, handling, and experimental diets, followed by 55 days of data recording.

The experiment followed a completely randomized design with five distinct dietary treatments, each comprising eight rams as experimental units. The control diet was formulated by including cashew nutshell liquid (CNSL) at 0.5% of the total dry matter (DM) combined with canola oil (added to 1.5% in total DM), a source predominantly rich in MUFA-omega-9 (ω-9 or n–9). The other four treatments consisted of the same CNSL inclusion level (0.5% DM) combined with different vegetable oils characterized by a high content of PUFA-omega-3 or 6 (corn, soybean, sunflower, and cottonseed), also added at 1.5% of the total dietary DM.

### 2.2. Chemical Analysis of Ingredients and Diets

To evaluate the chemical composition of the diets, samples of silage, concentrate ingredients, and feed refusals were initially pre-dried in a forced-air oven at 55 °C for 72 h. After drying, the samples were ground to pass through a 1 mm sieve using a Wiley-type mill. The proximate composition was determined using standard procedures established by the Association of Official Analytical Chemists (AOAC) [25], which included the determination of dry matter (method 934.01), ash (method 942.05), crude protein (method 968.06), and ether extract (method 920.39).

Neutral detergent fiber (NDF) was analyzed according to the method described by Van Soest et al. [26], with corrections for non-fiber components, as outlined by Senger et al. [27]. The procedure employed thermostable α-amylase (Sigma A3306; Sigma-Aldrich, Steinheim, Germany), and the results were reported as ash-free NDF (aNDF). Lignin content was determined by treating the aNDF residue with 72% sulfuric acid using AOAC method 973.18 [25]. Neutral detergent-insoluble protein (NDIP) and acid detergent-insoluble protein (ADIP) were quantified following the protocol of Licitra et al. [28].

The non-fiber carbohydrate (NFC) content was estimated using the equation proposed by Mertens [29], which incorporates NDF values corrected for ash and crude protein. Total digestible nutrients (*TDN*) in the diet were calculated based on digestible fractions using the approach reported by Andrade et al. [11]:

(1) TDN (g/kg)=(TDN intake ÷ DM intake)×100

Metabolizable energy (*ME*) was estimated using the method described by Weiss [30], where *TDN* values served as the basis for calculation. The *TDN* percentage was derived using Equation (2) [11]:

(2) TDN (%)=% digestible CP+% digestible NDF+(% digestible EE×2.25)+% digestible NFC

Digestible energy (*DE*) was obtained by converting *TDN* values using the factor of 4409 kcal *DE* per kg of *TDN*, and the metabolizable energy (*ME*) was then estimated from Equation (3):

(3) ME=DE×0.82

### 2.3. Slaughter and Collection of the Longissimus Lumborum Muscle

At the end of the experimental period, all rams were weighed in a commercial slaughterhouse 1.0 mile away from the feedlot, using an electronic scale to record slaughter body weight after a 16 h fasting period. Subsequently, the animals were stunned via electrical stunning (minimum current of 1.25 amperes) using appropriate equipment (Dal Pino, Santo André, SP, Brazil). Exsanguination was performed by severing the jugular veins and carotid arteries, followed by skinning, evisceration, removal of the head at the atlanto-occipital joint, and removal of the distal limbs at the metacarpophalangeal and metatarsophalangeal joints. The left and right *longissimus lumborum* (LL) muscles were dissected, vacuum-packed, properly labeled, and stored at −20 °C for subsequent physicochemical composition analysis.

### 2.4. Physicochemical Composition and Proximate Analysis of the Longissimus Lumborum Muscle

The pH of the *longissimus lumborum* muscle was evaluated approximately 45 min after slaughter (initial pH) and again at 24 h postmortem (ultimate pH) following carcass chilling. Measurements were obtained from the left side of each carcass between the 12th and 13th ribs using a portable penetration pH meter (Testo Instrument Co., LTD., Schwarzwald, Germany), which was calibrated before use with standard buffer solutions of pH 4.0 and 7.0, as recommended by the manufacturer.

Meat color was assessed immediately after pH determination on the freshly exposed surface of the *longissimus lumborum*. To allow myoglobin oxygenation, the samples were exposed to air at a temperature of 6–7 °C for 40 min [31]. Color attributes [lightness (L*), redness (a*), and yellowness (b*)] were measured using the CIE color system [32] with a Minolta Chroma Meter CR-400 (Minolta Camera Co., Osaka, Japan). Before measurement, the device was calibrated with standard white and black reference tiles. Each sample was measured in triplicate, and the mean values were recorded for analysis.

Water-holding capacity (WHC) was evaluated by placing approximately 300 mg of muscle between pre-weighed filter papers (Albert 238, 12.5 cm diameter) and applying a constant pressure of 3.6 kg for 5 min [33]. WHC was calculated as the percentage of water retained, based on the difference in filter paper weight before and after compression, and expressed as g/100 g of muscle.

Cooking loss (CL) was determined using the American Meat Science Association (AMSA) guidelines [34]. Two subsamples of meat, each approximately 2.5 cm thick and free from visible subcutaneous fat, were individually weighed and cooked on an electric grill (George Foreman^®^ Jumbo Grill GBZ6BW, Rio de Janeiro, Brazil). A stainless-steel thermocouple (Gulterm 700; Gulton do Brazil, Sao Paulo, Brazil) was inserted into the geometric center of each sample to monitor the internal temperature, which was maintained until it reached 71 °C. After cooking, the samples were allowed to cool to room temperature and reweighed, and the cooking loss was calculated as the percentage of weight lost during thermal processing.

Following CL assessment, three cylindrical core samples (1.0 cm in diameter × 2.0 cm in length) were extracted from each steak toward muscle fiber orientation for shear force analysis [34]. Warner–Bratzler shear force (WBSF) was measured using a texture analyzer (TX-TX2, Mecmesin, NV, USA) equipped with a Warner–Bratzler blade. The device was operated at a crosshead speed of 20 cm/min with a 25 kg/f load, and cuts were made perpendicular to the muscle fibers. Shear force results were expressed in Newtons (N), according to the protocol of the Meat Animal Research Center [35].

For proximate composition, samples of the *longissimus lumborum* were lyophilized for 72 h, finely ground, and analyzed for moisture content (AOAC 967.03), crude protein (AOAC 981.10), ether extract (AOAC 960.39), and ash (AOAC 942.05), following the official methods of the Association of Official Analytical Chemists [25].

### 2.5. Fatty Acid Determinations

The FA composition of the diets (Table 2) was assessed following a modified version of the procedure outlined by Palmquist and Jenkins [36]. Two replicate portions, each containing 0.5 g of dried sample, were transferred into Pyrex test tubes fitted with Teflon-lined screw caps. Then, 2 mL of hexane and 3 mL of a 10% methanolic solution of acetyl chloride were added to each tube for lipid extraction and methylation. The tubes were vortexed (Fisatom 772, São Paulo, Brazil), then heated in a water bath at 90 °C for 2 h and subsequently cooled. After cooling, 1 mL of hexane and 10 mL of 6% K_2_CO_3_ were added. The samples were vortexed again, then centrifuged (Centribio 80–2B, Equipar Ltd., Paraná, Brazil) for 5 min. The upper (organic) phase was collected and transferred to tubes containing 1.0 g of Na_2_SO_4_ and activated charcoal for drying. Following a second centrifugation, the supernatant was transferred to chromatographic vials for gas chromatography (GC) analysis.

Fatty acid methyl esters (FAMEs) were separated using a gas chromatograph (Perkin Elmer Clarus 680, Waltham, MA, USA) equipped with a flame ionization detector (FID) and a fused silica capillary column ELITE-WAX (30 m × 0.32 mm × 0.25 µm). Analytical conditions were as follows: injector at 250 °C, detector at 280 °C, and column temperature initially set at 150 °C for 16 min, ramped at 2 °C/min to 180 °C (held for 25 min), then increased at 5 °C/min to 210 °C (held for 25 min). Helium was used as the carrier gas at a flow rate of 1 mL/min; hydrogen and synthetic air were supplied at 30 mL/min and 300 mL/min, respectively. The injection volume was 1 µL in duplicate per sample. Fatty acids were identified by comparing retention times to a standard FAME mixture (Sigma-Aldrich, Saint Louis, MO, USA, catalog no. 189–19), and the results were expressed as g/100 g of total FAME based on peak area normalization.

The fatty acid profile of the *longissimus lumborum* muscle was determined using a modified version of the method described by O’Fallon et al. [37]. Approximately 0.5 g of lyophilized tissue was weighed into Pyrex screw-cap tubes (16 × 125 mm), to which 0.7 mL of 10 N potassium hydroxide (KOH), 5.3 mL of methanol, and 1.0 mL of an internal standard solution (1.0 mg C19:0 per mL methanol) were added. The tubes were incubated in a water bath at 55 °C for 90 min, with manual agitation every 20 min for 5 s. After incubation, the tubes were cooled under running water, followed by the 0.58 mL of 24 N sulfuric acid (H_2_SO_4_). The heating step was then repeated under the same conditions.

After the reaction, 3.0 mL of hexane was added to each tube. The mixtures were vortexed (Fisatom 772, São Paulo, Brazil) for 90 s and centrifuged (Centribio 80–2B, Equipar Ltd., Paraná, Brazil) to separate the phases. The upper (hexane) layer containing the fatty acid methyl esters (FAMEs) was carefully collected and transferred to chromatographic vials for subsequent analysis.

The FAMEs were separated using a gas chromatograph (Focus GC 180, Thermo Electron SPA, Milan, Italy) fitted with a SUPELCO SP™–2560 capillary column (100 m × 0.25 mm × 0.20 µm; Supelco Inc., Bellefonte, PA, USA) and a flame ionization detector. The injector temperature was set at 250 °C, and the detector at 280 °C. A split injection mode was used with a ratio of 30:1, and the injection volume was 1 µL. The oven temperature was initially programmed at 140 °C, then increased at 1 °C per minute until it reached 220 °C, where it was held for 25 min. Hydrogen served as the carrier gas, with a 1.5 mL/min flow rate. Fatty acid identification was based on a comparison with the retention times of a standard mixture containing 52 known fatty acids (GLC-674, Nu-Chek Prep, Inc., Elysian, MN, USA). Quantification followed the method proposed by Sukhija and Palmquist [38], and the results were expressed as milligrams per 100 g of lyophilized sample.

From the resulting fatty acid data, the total contents of saturated (ΣSFA), monounsaturated (ΣMUFA), and polyunsaturated (ΣPUFA) fatty acids were calculated. The ratios ΣMUFA:ΣSFA, ΣPUFA:ΣSFA, ΣPUFA:ΣMUFA, and Σn–6:Σn–3 were also determined. The nutritional quality indices of the lipid fraction, including the atherogenicity index (AI) and thrombogenicity index (TI), were calculated according to the equations by Ulbricht and Southgate [39]. The hypocholesterolemic to hypercholesterolemic fatty acid ratio (h:H) was determined based on the criteria of Santos-Silva et al. [40], and the desirable fatty acid (DFA) content was estimated using the method proposed by Rhee [41]. Enzymatic activity indices, including stearoyl-CoA desaturase (Δ9-desaturase) activity for palmitic acid (C16:0) and stearic acid (C18:0), as well as elongase activity, were calculated using the equations suggested by Smet et al. [42].

### 2.6. Statistical Analysis

The data were analyzed using a randomized complete block design through the MIXED procedure in SAS software (SAS Institute Inc., 9.1 version, Cary, NC, USA) [43]. Each animal served as the experimental unit for all measured variables. The statistical model applied is expressed by Equation (4):

(4) Yijk=µ+Oj+eijk

wherein *Yij* = value referring to the observation of repetition *i* of treatment *j*; µ = overall mean; *Oj* = effect of treatment *j* [*j* = 0.5% of CNSL + 1.5% canola oil (MUFA) or CNSL + 1.5% soybean oil (PUFA) or CNSL + 1.5% corn oil (PUFA) or CNSL + 1.5% cottonseed oil (PUFA) or CNSL + 1.5% sunflower oil (PUFA)]; *eij* = random error associated with the observation. Treatment means (*n* = 10) were compared using Tukey’s multiple comparison test, with significance set at *p* < 0.05.

## 3. Results

The different FA composition from the CNSL and oils blend did not affect (*p* > 0.05) initial pH (45 min postmortem) and the lightness (L*) and redness (a*) color indexes (Table 3). However, the pH at 24 h postmortem was higher for the combination of CNSL + canola oil (MUFA n–9) compared to corn oil (PUFA), with no significant difference from the other vegetable oils (*p* < 0.05). Water-holding capacity (WHC) was higher and cooking loss was lower in the meat of mutton fed with CNSL + canola (MUFA-n–9) compared to diets containing CNSL + PUFA-rich vegetable oils (sunflower and soybean) (*p* < 0.05). Rams fed with CNSL + canola (MUFA) produced softer meat, that is, with lower shear force compared to those fed with CNSL + PUFA-rich vegetable oils, regardless of the specific oil used (*p* < 0.05). Yellowness (b*) was more intense in the meat of mutton fed with a blend of CNSL + cottonseed or soybean compared to CNSL + corn oil, with no significant difference from the other vegetable oils (*p* < 0.05).

There was no effect (*p* > 0.05) of mixing different vegetable oils with CNSL on the proximate composition (moisture, crude protein, crude fat, or crude ash) of the mutton meat (Table 4).

The different FA composition of the CNSL and oil blends did not change (*p* > 0.05) the concentrations of SFAs C4:0, C10:0, C12:0, C15:0, C16:0, C17:0, C18:0 and C20:0; MUFAs C14:1, C15:1, C17:1, and C18:1 c–9; or PUFAs C18:2 n–6, C20:2, C20:3 n–6, and C20:4 n–6 in the *longissimus lumborum* muscle of mutton (Table 5). However, the SFA C14:0 and MUFA C16:1 presented higher concentrations in mutton fed with a combination of CNSL + canola oil (MUFA-n–9) and CNSL + cottonseed oil (PUFA) compared to CNSL + corn, with no significant differences among the other vegetable oils (*p* < 0.05).

In contrast, rams fed with CNSL + canola, cottonseed, or corn oils presented higher concentrations of C18:2 t–10,12 and C18:3 n–3 compared to CNSL + soybean oil (*p* < 0.05). The concentration of PUFA C20:5 n–3 (EPA) was higher in the *longissimus lumborum* muscle of mutton fed with a blend of CNSL + soybean oil compared to CNSL + canola or cottonseed or corn oils (*p* < 0.05). CNSL + sunflower oil also presented a lower EPA concentration than all vegetable oils. The DHA (C22:6 n–3) presented a higher concentration in the muscle of rams fed CNSL + cottonseed and a lower concentration in rams fed a CNSL + soybean oil diet (*p* < 0.05).

The different UFA diets did not change (*p* > 0.05) ΣSFA, ΣMUFA, ΣPUFA, ∑n–9 ∑n–6 ∑n–3 and the proportions ΣMUFA:ΣSFA and ΣPUFA:ΣSFA in the *longissimus lumborum* muscle of mutton (Table 6). However, rams fed with CNSL + soybean presented a lower ∑n–3 compared to other vegetable oils and a lower ∑n–6:∑n–3 ratio compared to rams fed with CNSL + cottonseed, which presented the highest ratio, with no difference among the other oils (*p* < 0.05).

The AI, TI, and h:H health indices, as well as the Δ9–desaturase C16 and C18 activity indices, were not affected (*p* > 0.05) by diets with different vegetable oils (source MUFA and PUFA) and CNSL blend (Table 6). However, the DFA and elongase enzyme activity presented higher concentrations in rams fed CNSL + soybean sunflower or corn compared to CNSL + canola oil (MUFA-n–9) and CNSL + cottonseed oil (*p* < 0.05).

## 4. Discussion

The lack of significant effects of the fatty acid (FA) composition of vegetable oil blends with cashew nutshell liquid (CNSL) on initial pH and the color parameters L* (lightness) and a* (redness) suggests that, in the immediate postmortem period, these attributes are significantly influenced by factors such as pre-slaughter management, initial anaerobic glycolysis, and the physiological status of the animals rather than by dietary lipid composition. Final pH values (after 24 h of chilling) decreased from an average of 6.49 to 5.52 across all treatments, remaining within the acceptable range for lamb meat [44]. This decline indicates that muscle glycogen reserves are adequate to sustain postmortem glycolysis, allowing for the proper conversion of muscle into meat and ensuring high meat quality [45,46,47].

Nevertheless, the higher ultimate pH observed in animals supplemented with CNSL combined with canola oil (rich in MUFA) compared to those fed CNSL with corn oil (rich in PUFA) suggests a metabolic effect associated with the type of fatty acid influencing muscle biochemistry and the rate of postmortem acidification. According to Kurma et al. [48], higher pH levels are associated with increased lipid oxidation. This could be attributed to the lower antioxidant capacity of canola oil, which is richer in MUFA (n–9) compared to PUFA-rich oils [49]. Despite the differences in pH after 24 h among treatments, the values remained within the normal range for sheep meat (5.5 to 5.8), as reported by Malva et al. [50].

From the perspective of ruminal biohydrogenation, MUFAs and PUFAs follow distinct metabolic pathways. PUFAs, such as linoleic acid (C18:2 n–6) and alpha-linolenic acid (C18:3 n–3), undergo extensive biohydrogenation, resulting primarily in stearic acid (C18:0) as the product [51]. This SFA decreases membrane fluidity, leading to more rigid cell membranes. In contrast, MUFAs, particularly oleic acid (C18:1 n–9), are more resistant to complete biohydrogenation, especially in the presence of phenolic compounds from CNSL, which inhibit lipolytic and biohydrogenating ruminal microorganisms [52]. Consequently, a greater proportion of MUFAs escapes ruminal hydrogenation and becomes available for intestinal absorption and tissue deposition.

A higher proportion of MUFAs in muscle directly influences the fluidity of the lipid matrix and its interaction with the protein matrix within the muscle tissue. MUFAs, like oleic acid, possess a single double bond that provides an optimal balance between fluidity and structural stability in intramuscular fat [53]. This property allows lipids to more effectively occupy interfibrillar and interfascicular spaces, acting as “biological lubricants” that reduce myofibrillar protein contraction during cooking [54]. As a result, water loss is minimized, which explains the increased water-holding capacity (WHC) and reduced cooking loss (CL) in meat from animals fed CNSL combined with MUFA-rich oils. Conversely, PUFAs have more kinked and flexible chains that form less cohesive lipid structures, thus limiting their ability to stabilize the muscle’s extracellular spaces. This reduces their capacity to form an effective lipid barrier to retain water within muscle tissues [55].

Furthermore, a higher intake of MUFAs may enhance cellular membrane fluidity, maintaining a more dynamic and flexible lipid structure. This directly impacts meat tenderness, as observed in lambs fed CNSL combined with canola oil. Muscle tissues with higher MUFA content tend to exhibit lower lipid melting points, facilitating the breakdown of structural proteins and lipids during cooking, which ultimately results in more tender meat [56,57]. In contrast, meat from PUFA-rich diets, while offering nutritional benefits to consumers, tends to develop lipid structures that are more prone to oxidation, negatively affecting oxidative stability, color, and water retention [58,59].

The more pronounced yellow color (higher b* values) observed in meat from animals supplemented with CNSL combined with cottonseed or soybean oils may be attributed to the greater deposition of lipophilic pigments such as carotenoids present in these oils or to the higher proportion of unsaturated fatty acids, which promote lipid oxidation and the formation of chromophoric compounds [60]. Such color changes may be perceptible to consumers and influence the sensory evaluation of lamb meat.

Lightness (L*) and redness (a*) values remained within acceptable ranges for lamb meat, whereas b* values showed a slight decline. For lamb, the acceptable ranges are L* between 31.4 and 40.0, a* between 12.27 and 20.0, and b* between 3.34 and 5.65, which are critical parameters for the overall quality classification of lamb meat [61,62]. The results also indicate that CNSL supplementation increased meat redness, corroborating the findings of Araújo et al. [21]. This effect is likely related to the dark red pigmentation formed during CNSL polymerization, primarily due to the presence of cardol [63,64]. Moreover, the antioxidant properties derived from the technical processing of CNSL are expected to influence meat redness by delaying lipid oxidation [20,21]. However, the consistent inclusion rate of CNSL (0.5%) across treatments may explain the absence of significant differences in redness among treatments.

The absence of treatment effects on the proximate composition of the meat (moisture, crude protein, ether extract, and ash) reinforces that the dietary lipid inclusion level, while sufficient to alter intramuscular fat quality and physicochemical properties, was not high enough to affect the absolute levels of these components. This outcome reflects the uniform energy intake among treatments, which did not lead to differences in animal performance, as previously reported by Andrade et al. [11]. Consequently, carcass fat percentages remained similar, with both body fat and moisture closely associated with the carcass finishing degree [59,65].

The lack of significant effects of the different CNSL and vegetable oil blends on most SFAs, MUFAs, and PUFAs indicates that the ruminal system has a high metabolic buffering capacity, particularly through the ruminal biohydrogenation process. This process is responsible for saturating unsaturated fatty acids and protecting ruminal microorganisms from the toxic effects of free fatty acids, especially PUFAs [14,17].

However, the significant increase in C14:0 and C16:1 in the muscle of the animals fed diets containing CNSL combined with canola oil (rich in MUFA-n–9) and cottonseed oil (rich in PUFA-n–6) suggests that part of the unsaturated fatty acids escaped complete biohydrogenation. This phenomenon may be related to the presence of phenolic compounds in CNSL, especially cardol and anacardic acid, which exert inhibitory effects on biohydrogenating bacteria, partially modulating this process [63,64]. Consequently, the accumulation of biohydrogenation intermediates, such as trans isomers and certain MUFA, is favored.

The higher concentrations of C18:2 t–10,12 and C18:3 n–3 in mutton fed with CNSL combined with canola, cottonseed, or corn oils compared to soybean oil indicate that the lipid profiles of these oils were more effective in modulating the ruminal microbiota, allowing greater intestinal flow of long-chain PUFAs. Conversely, soybean oil, which is rich in linoleic acid (C18:2 n–6), appears to have undergone a greater extent of biohydrogenation, resulting in lower tissue deposition of n–3 PUFA and higher conversion into saturated intermediates.

The significantly higher concentration of EPA (C20:5 n–3) in the muscle of animals fed CNSL + soybean oil can be explained by the elongation and desaturation pathway of linolenic acid (C18:3 n–3) in animal tissues, despite this pathway being limited in ruminants. However, the lower DHA (C22:6 n–3) concentration observed in animals fed CNSL + soybean oil suggests that although EPA synthesis occurred, the subsequent conversion to DHA was limited, likely due to the low activity of Δ6-desaturase and elongase enzymes, as well as the metabolic competition between n–6 and n–3 FA [17,50].

Despite these specific changes, no significant differences were observed in the total sums of ΣSFA, ΣMUFA, and ΣPUFA or the ratios ΣMUFA:ΣSFA and ΣPUFA:ΣSFA. This supports the hypothesis that ruminal biohydrogenation and hepatic lipid metabolism possess robust homeostatic mechanisms to maintain tissue lipid balance, even when exposed to diets with varying degrees of unsaturation [56,62].

Nevertheless, rams fed with CNSL + soybean oil showed lower ∑n–3 and ∑n–6:∑n–3 ratio compared to ram fed CNSL + cottonseed oil, which presented the highest ratio. The ∑n–6:∑n–3 ratio is a key nutritional indicator related to human health, as lower ratios are associated with reduced risks of cardiovascular, inflammatory, and metabolic diseases [66]. Therefore, from this perspective, the meat from animals fed soybean oil exhibited a healthier lipid profile.

The lipid health indices (AI, TI, and h:H ratio) were not affected by the different oil blends, indicating that variations in individual fatty acids were not sufficient to significantly alter the potential cardiovascular health risks associated with consuming this meat. On the other hand, the increased elongase enzyme activity and desirable fatty acid (DFA) index observed in animals fed CNSL combined with soybean, corn, or sunflower oils suggest an enhanced capacity for fatty acid chain elongation, promoting the formation of long-chain fatty acids such as EPA. Elongase enzymes convert C18 FA into C20 and C22 derivatives, which are critical biochemical steps in the biosynthesis of long-chain omega-3 fatty acids (EPA and DHA) [17,50].

From a chemical perspective, the difference between MUFAs and PUFAs directly impacts the oxidative stability and metabolic fate of lipids in meat. MUFAs, with a single double bond, exhibit greater oxidative stability compared to PUFAs, which contain two or more double bonds and are therefore more prone to lipid peroxidation. However, PUFAs are biologically more valuable for human health due to their anti-inflammatory, hypocholesterolemic, and cardioprotective properties [45,61].

## 5. Conclusions

In this study, dietary supplementation with different vegetable oils influenced meat quality attributes in the *longissimus lumborum* muscle of the rams, primarily through variations in monounsaturated (MUFA) and polyunsaturated (PUFA) fatty acid profiles, modulated by ruminal biohydrogenation and the antioxidant properties of phenolic compounds from cashew nutshell liquid (CNSL). Canola oil (MUFA content) improved sheep meat tenderness, juiciness, and water-holding capacity and reduced cooking losses compared to PUFA-rich sources. Additionally, combining oils helped reduce the astringency typically associated with CNSL, confirming the efficacy and safety of including it at 0.5% of the total diet. These findings suggest that selecting cost-effective oils rich in MUFAs can enhance meat quality and support the sustainable use of agro-industrial byproducts. However, the limitations of this study include the lack of long-term evaluation of animal performance and the absence of sensory analysis to validate consumer acceptance of the final product.

## Figures and Tables

**Table 1:** Proportion of ingredients and chemical composition of experimental diets.

Item	Vegetables Oils + CNSL ^1^ Blend				
	Soybean	Cottonseed	Sunflower	Corn	Canola
Ingredients proportions					
Sorghum silage	40.0	40.0	40.0	40.0	40.0
Ground corn	40.0	40.0	40.0	40.0	40.0
Soybean meal	16.5	16.5	16.5	16.5	16.5
CNSL ^1^	0.50	0.50	0.50	0.50	0.50
Vegetables oil	1.50	1.50	1.50	1.50	1.50
Mineral mixture	1.50	1.50	1.50	1.50	1.50
Chemical composition of the diet (g/kg)					
Dry matter	694	694	694	694	694
Ash	51.9	51.9	51.9	51.9	51.9
Crude protein	135	135	135	135	135
Ether extract	58.0	58.0	58.0	58.0	58.0
aNDF ^2^	371	371	371	371	371
Non-fiber carbohydrates	384	384	384	384	384
Total digestible nutrients	792	794	755	723	782
Metabolizable energy ^3^	2.86	2.87	2.73	2.61	2.83
Chemical composition of the effective intake diet (g/kg DM)					
Crude protein	113	118	118	112	114
Ash	51.9	52.8	50.5	54.3	49.8
aNDF ^2^	392	378	392	390	362
Ether extract	41.9	47.7	45.0	47.4	44.7
Non-fiber carbohydrates	400	403	394	396	429
Total digestible nutrients	760	767	764	764	768
Metabolizable energy ^3^	3.79	3.95	4.01	4.00	3.79

^1^ Cashew nutshell liquid; ^2^ Neutral detergent fiber assayed with a heat stable amylase and expressed exclusive for residual ash; ^3^ Calculated based on Andrade et al. [11].

**Table 2:** Composition of the fatty acids identified in the vegetable oils and CNSL in the diets.

Fatty Acids (g/100 g FAME)		Vegetable Oils and CNSL ^1^ Blend					
		MUFA	PUFA				
		Canola	Cottonseed	Sunflower	Corn	Soybean	CNSL ^1^
Myristic	C14:0	0.18	0.50	0.33	0.13	0.09	1.77
Palmitic	C16:0	2.52	21.7	8.54	11.7	12.2	15.9
Stearic	C18:0	2.30	2.10	7.44	5.17	3.27	5.76
Palmitoleic	C16:1	0.20	0.40	-	0.10	-	8.22
Oleic	C18:1	63.3	17.2	25.7	20.7	29.4	14.3
Linoleic	C18:2	23.7	50.3	54.2	53.9	45.8	10.8
Linolenic	C18:3	8.8	2.41	0.32	5.51	3.78	2.83
Other FA ^2^	-	-	5.40	3.47	2.70	5.46	40.4
Saturated	SFA	5.00	24.3	16.23	17.0	15.6	23.5
Monounsaturated	MUFA	63.5	17.6	25.7	20.8	29.4	22.5
Polyunsaturated	PUFA	31.5	52.7	54.5	59.5	49.6	13.7
Unsaturated	UFA	94.5	70.3	80.2	80.3	79.0	36.2

^1^ Cashew nutshell liquid at 1.5% of total dry matter; vegetables oils at 1.5% of total dry matter: Canola = Source of monounsaturated fatty acids (MUFAs); Cottonseed, sunflower, corn, and soybean = Source of polyunsaturated fatty acids (PUFAs); ^2^ Sum of fatty acids whose peaks could not be identified in the analysis or did not exist.

**Table 3:** Meat quality of rams fed diets containing mixtures of cashew nutshell liquid (CNSL; 0.5% of total DM) and different vegetable oils (1.5% of total DM).

Variable	Vegetable Oils and CNSL ^1^ Blend					SEM ^2^	*p*-Value ^3^
	MUFA	PUFA					
	Canola	Cottonseed	Sunflower	Corn	Soybean		
Initial pH (45 min)	6.58	6.58	6.43	6.42	6.45	0.06	0.102
Final pH (24 h)	5.59 ^a^	5.51 ^ab^	5.50 ^ab^	5.47 ^b^	5.55 ^ab^	0.02	0.016
Water-holding capacity (%)	31.7 ^a^	30.5 ^ab^	28.8 ^b^	30.1 ^ab^	28.7 ^b^	1.32	0.047
Cooking loss (%)	26.5 ^b^	31.0 ^ab^	32.1 ^a^	28.3 ^ab^	32.3 ^a^	1.27	0.006
Shear force (N)	15.1 ^b^	17.1 ^a^	17.4 ^a^	17.8 ^a^	17.5 ^a^	0.13	0.036
Lightness (L*)	38.8	39.3	39.3	38.5	39.1	0.43	0.418
Redness (a*)	19.9	20.1	20.2	19.6	19.6	0.19	0.117
Yellowness (b*)	1.72 ^ab^	1.86 ^a^	1.59 ^ab^	1.22 ^b^	2.01 ^a^	0.13	0.003

^1^ Cashew nutshell liquid at 1.5% of total dry matter; vegetables oils at 1.5% of total dry matter: Canola = Source of monounsaturated fatty acids (MUFA); Cottonseed, sunflower, corn, and soybean = Source of polyunsaturated fatty acids (PUFAs); ^2^ SEM = Standard error of the mean; ^3,ab^ Means followed by a different letter in the row are statistically different according to Tukey’s test at 5% probability.

**Table 4:** Proximate composition of *longissimus lumborum* of the mutton fed diets containing mixtures of cashew nutshell liquid (CNSL; 0.5% of total DM) and different vegetable oils (1.5% of total DM).

Item (g/100 g Meat)	Vegetable Oils and CNSL ^1^ Blend					SEM ^2^	*p*-Value ^3^
	MUFA	PUFA					
	Canola	Cottonseed	Sunflower	Corn	Soybean		
Moisture	71.6	71.2	71.1	72.2	71.3	0.44	0.110
Crude protein	23.6	24.6	24.7	23.5	24.2	0.34	0.210
Crude fat	3.59	3.01	2.86	2.86	3.23	0.16	0.141
Crude ash	1.23	1.17	1.33	1.44	1.28	0.08	0.248

^1^ Cashew nutshell liquid at 1.5% of total dry matter; vegetables oils at 1.5% of total dry matter: Canola = Source of monounsaturated fatty acids (MUFAs); Cottonseed, sunflower, corn, and soybean = Source of polyunsaturated fatty acids (PUFAs); ^2^ SEM = Standard error of the mean. ^3^ Means followed in the row are statistically different according to Tukey’s test at 5% probability.

**Table 5:** Fatty acid composition (mg/100 g fresh meat) of the *longissimus lumborum* muscle from mutton fed diets containing mixtures of cashew nutshell liquid (CNSL; 0.5% of total DM) and different vegetable oils (1.5% of total DM).

Fatty Acids (mg/100 g Muscle)	Vegetable Oils and CNSL ^1^ Blend					SEM ^2^	*p*-Value ^3^
	MUFA	PUFA					
	Canola	Cottonseed	Sunflower	Corn	Soybean		
Saturated fatty acids (SFA)							
C4:0	0.69	0.70	0.53	0.61	0.58	0.33	0.9754
C10:0	2.89	3.05	2.91	2.61	2.97	0.31	0.5933
C12:0	1.89	2.34	1.99	1.99	2.15	0.54	0.7695
C14:0	46.9 ^a^	48.13 ^a^	43.65 ^ab^	38.9 ^b^	43.0 ^ab^	1.34	0.0312
C15:0	5.30	5.05	5.09	4.55	5.45	0.313	0.3367
C16:0	573.3	579.4	538.2	512.6	538.4	19.75	0.1236
C17:0	18.4	17.1	19.0	16.3	18.4	1.13	0.4834
C18:0	364.3	380.7	346.6	351.8	368.8	12.3	0.3210
C20:0	47.2	51.1	54.8	51.3	53.3	5.26	0.8777
Monounsaturated fatty acids (MUFA)							
C14:1	1.31	1.50	1.20	1.13	1.09	0.21	0.6971
C15:1	9.72	11.5	8.01	9.67	10.55	1.06	0.2250
C16:1	42.43 ^a^	42.1 ^a^	39.2 ^ab^	35.0 ^b^	37.5 ^ab^	1.41	0.0191
C17:1	11.3	10.7	9.40	10.2	11.2	0.80	0.397
C18:1 c–9	1078.3	1099.7	1027.5	1032.5	1084.7	41.3	0.652
Polyunsaturated fatty acids (PUFA)							
C18:2 n–6	100.5	82.25	92.3	109.1	105.5	8.20	0.162
C18:2 t–10 t–12 ^4^	2.55 ^b^	2.88 ^ab^	3.06 ^a^	2.52 ^b^	3.11 ^a^	0.11	0.007
C18:3 n–3	5.74 ^b^	5.51 ^b^	6.48 ^ab^	5.97 ^b^	8.74 ^a^	0.59	0.003
C20:2	4.69	6.31	5.19	6.38	5.96	0.78	0.486
C20:3 n–6	2.15	3.56	1.29	1.80	2.132	0.63	0.156
C20:4 n–6	30.96	26.68	23.9	31.7	34.51	2.61	0.052
C20:5 n–3	3.95 ^b^	3.32 ^b^	1.91 ^c^	3.66 ^b^	5.03 ^a^	0.68	0.046
C22:6 n–3	0.97 ^ab^	1.43 ^a^	0.97 ^ab^	0.68 ^b^	0.28 ^c^	0.35	0.028

^1^ Cashew nutshell liquid at 1.5% of total dry matter; vegetables oils at 1.5% of total dry matter: Canola = Source of monounsaturated fatty acids (MUFA); Cottonseed, sunflower, corn, and soybean = Source of polyunsaturated fatty acids (PUFAs); ^2^ SEM = Standard error of the mean. ^3,abc^ Means followed by a different letter in the row are statistically different according to Tukey’s test at 5% probability; ^4^ CLA, conjugated linoleic acid.

**Table 6:** Fatty acid group, sum (Ʃ), ratios, and health indexes (mg/100 g fresh meat) in the *longissimus lumborum* muscle of mutton fed diets containing mixtures of cashew nutshell liquid (CNSL; 0.5% of total DM) and different vegetable oils (1.5% of total DM).

Fatty Acids ^4^ (g/100 g FAME)	Vegetable Oils and CNSL ^1^ Blend					SEM ^2^	*p*-Value ^3^
	MUFA	PUFA					
	Canola	Cottonseed	Sunflower	Corn	Soybean		
Sum							
∑SFA	45.0	44.2	44.1	43.9	43.9	0.525	0.568
∑MUFA	48.2	48.1	49.2	48.4	48.8	0.885	0.912
∑PUFA	6.69	7.56	6.78	7.63	7.307	0.701	0.852
∑n–9	48.2	48.1	49.2	48.4	48.8	0.885	0.912
∑n–6	1.27	1.54	1.21	1.47	1.56	0.145	0.354
∑n–3	0.45 ^b^	0.43 ^b^	0.43 ^b^	0.48 ^b^	0.64 ^a^	0.054	0.037
Ratio							
∑MUFA:∑SFA	1.07	1.09	1.12	1.10	1.11	0.029	0.807
∑PUFA:∑SFA	0.148	0.141	0.151	0.160	0.165	0.012	0.711
n–6:n–3	2.91 ^ab^	3.93 ^a^	2.72 ^ab^	2.94 ^ab^	2.47 ^b^	0.297	0.017
Health indexes							
IA	0.64	0.64	0.64	0.59	0.59	0.018	0.102
IT	1.57	1.58	1.54	1.50	1.46	0.040	0.209
h:H	1.73	1.76	1.81	1.87	1.87	0.046	0.137
DFA	70.4 ^b^	70.7 ^b^	71.8 ^a^	71.7 ^a^	71.7 ^a^	0.289	0.049
Enzymatic activity							
Δ9–desaturase C16	6.86	6.75	7.07	6.38	6.54	0.261	0.401
Δ9–desaturase C18	74.6	74.2	74.2	74.5	74.6	0.831	0.988
Elongase	70.0 ^b^	70.4 ^b^	71.0 ^a^	71.6 ^a^	71.6 ^a^	0.225	0.043

^1^ Cashew nutshell liquid at 1.5% of total dry matter; vegetables oils at 1.5% of total dry matter: Canola = Source of monounsaturated fatty acids (MUFAs); Cottonseed, sunflower, corn, and soybean = Source of polyunsaturated fatty acids (PUFAs); ^2^ SEM = Standard error of the mean. ^3,ab^ Means followed by a different letter in the row are statistically different according to Tukey’s test at 5% probability. ^4^ SFA, saturated fatty acid; ∑MUFA, sum of monounsaturated fatty acids; ∑PUFA, sum of polyunsaturated fatty acids; n–9, omega-9 PUFA; n–6, omega-6 PUFA; n–3, omega-3 PUFA; h/H, hypocholesterolemic and hypercholesterolemic fatty acid ratio; IA, atherogenicity index; TI, thrombogenicity index; DFA, desirable fatty acid.

## Data Availability

The data that support the findings of this study are available from the corresponding author upon reasonable request.

## Abbreviations

The following abbreviations are used in this manuscript:

*a**	redness
ADF	acid detergent fiber
AI	atherogenic index
*b**	yellowness
CLA	conjugated linoleic acid
CP	crude protein
CWL	cooking weight loss
DHA	docosahexaenoic acid
DFA	desirable fatty acids
DM	dry matter
EE	ether extract
EPA	eicosapentaenoic acid
FAME	fatty acid methyl esters
FA	fatty acid
h: H	hypocholesterolemic and hypercholesterolemic fatty acids ratio
*L**	lightness
MCFA	medium-chain-FA
MUFA	summed monounsaturated fatty acids
n–3	omega-3
n–6	omega-6
n–9	omega-9
NFC	nonfibrous carbohydrate
NDF	neutral detergent fiber
aNDF	Neutral detergent fiber assayed with a heat stable amylase and expressed exclusive for residual ash
PUFA	polyunsaturated fatty acids
SFA	saturated fatty acids
TI	thrombogenicity index
TDN	total digestible nutrients
UFA	total unsaturated fatty acids
WHC	water-holding capacity
